# Crop Photosynthetic Performance Monitoring Based on a Combined System of Measured and Modelled Chloroplast Electron Transport Rate in Greenhouse Tomato

**DOI:** 10.3389/fpls.2020.01038

**Published:** 2020-07-10

**Authors:** Wenjuan Yu, Oliver Körner, Uwe Schmidt

**Affiliations:** ^1^ Department of Biosystems Engineering, Humboldt University of Berlin, Berlin, Germany; ^2^ Next-Generation Horticultural Systems, Leibniz-Institute of Vegetable and Ornamental Crops (IGZ), Grossbeeren, Germany

**Keywords:** biochemical photosynthesis model, chlorophyll fluorescence, CO_2_ gas exchange, electron transport rate, photosynthesis, photosynthesis modelling, quantum yield

## Abstract

Combining information of plant physiological processes with climate control systems can improve control accuracy in controlled environments as greenhouses and plant factories. Through that, resource optimization can be achieved. To predict the plant physiological processes and implement them in control actions of interest, a reliable monitoring system and a capable control system are needed. In this paper, we focused on the option to use real-time crop monitoring for precision climate control in greenhouses. For that, we studied the processes and external factors influencing leaf net CO_2_ assimilation rate (*A_L_*, µmol CO_2_ m^-2^ s^-1^) as possible variables of a plant performance indicator. While measured greenhouse environmental variables such as light, temperature, or humidity showed a direct relation between *A_L_* and light-quantum yield of photosystem II (Φ_2_), we defined three objectives: (1) to explore the relationship between climate variables and *A_L_*, as well as Φ_2_; (2) create a simple and reliable method for real‐time prediction of *A_L_* with continuously Φ_2_ measurements; and (3) calibrate parameters to predict chloroplast electron transport rate as input in *A_L_* modelling. Due to practical obstacles in measuring CO_2_ gas-exchange in commercial production, we explored a method to predict *A_L_* by measuring Φ_2_ of leaves in a commercial hydroponic greenhouse tomato crop (“Pureza”). We calculated *A_L_* with two different approaches based on either the negative exponential response model with simplified biochemical equations (marked as Model I) or the non-rectangular hyperbola full biochemical photosynthetic models (marked as Model II). Using Model I can only be used to predict *A_L_* with large uncertainty (R^2^ 0.64; RMSE 2.21), while using Φ_2_ as input to Model II could be used to improve the prediction accuracy of *A_L_* (R^2^ 0.71; RMSE 1.98). Our results suggests that (1) Φ_2_ light signals can be used to predict net photosynthesis rate with high accuracy; (2) a parameterized photosynthetic electron transport rate model is suitable predicting measured electron transport rate (*J*) and *A_L_*. The system can be used as decision support system (DSS) for plant and crop performance monitoring when leaf-dynamics are up-scaled to the plant or crop level.

## Introduction

In modern greenhouses and plant factories plant cultivation is usually done with computerized environmental climate control. To achieve the desired climate, a great variety of controllers and actuators are used (Körner and Van Straten, 2008; Rytter et al., 2012; Shamshiri et al., 2017; Gurban and Andreescu, 2018; Ramin et al., 2018), often supported by model-based decision support systems (DSS) (e.g. Körner, 2019). Although sensor-based monitoring and real-time model predictions strongly improved early warning and greenhouse climate control (Körner and Hansen, 2012; Mahlein, 2015; Körner, 2019), real-time crop monitoring still suffers from inadequate equipment and/or insufficiently model quality. The realization of soft-sensors (i.e. mathematical models using real-time sensor data) (De Koning, 2006) with deterministic explanatory models in greenhouse cultivation monitoring is still under development. In here, robust and simple sensors combined with models calibrated with data from laboratory experiments would be the most suitable approach to implement physiological based automatic control system in the greenhouse (Janka et al., 2013; Körner, 2019). To achieve that, a reliable system with both measured and modelled plant physiological parameters is needed.

Plant photosynthesis is a physiological process suitable to be used in DSS-development with monitoring and assessment tools. A monitoring system, initially based on measuring leaf net CO_2_ exchange (*A_L_*), was used as starting point in this study (BERMONIS, Steinbeis GmbH & Co. KG for Technology Transfer, Berlin, Germany). BERMONIS is real time photosynthesis monitoring system developed for long-time continuously measurement leaf gas exchange (Schmidt, 1998; Schmidt, 2005). The system can be used to up-scale multiple measured single leaf *A_L_* to crop photosynthesis (*A_crop_*, µmol CO_2_ m^-2^ s^-1^) by considering the variations of both light distribution and specific leaf photosynthetic capacity within the plants' canopy; e.g. Huber (2011) used BERMONIS in combination with psychrometric charts to detect and follow the “comfort zone” for an adult tomato crop in real-time.

Another widely used and accepted approach to measure plant photosynthetic productivity is chlorophyll fluorescence analysis (CFA). With the pulse amplitude modulation method of CFA (PAM), the light beams are modulated and the system detects fluorescence excited by the measuring light in the presence of background illumination (Schreiber, 1986; Schreiber et al., 1986; Govindjee, 1995; Schreiber, 2004; Baker, 2008; Tschiersch et al., 2017). Its small size, ease to transfer, and high sensitivity have made PAM-CFA a widely accepted method for plant stress detection (Lawlor, 1995; Cornic and Massacci, 1996; Flexas et al., 1998; Flexas et al., 2000; Lawlor and Cornic, 2002). In comparison to the gas exchange method used for plant photosynthetic productivity measurements, CFA is more sensitive to plant water deficit: Water deficit leads to closed stomata that limits CO_2_ uptake, followed by reduced energy use and excessive light energy absorption. This results in an activated protection mechanism and increase of non-photochemical quenching (NPQ), which is one of the main variables used in CFA. This process is commonly faster than gas exchange (Herppich et al., 1996; Herppich and Peckmann, 1997; Herppich and Peckmann, 2000). Therefore, it is of great practical significance to apply CFA parameters to simulate the CO_2_ assimilation of plant leaves (Krall and Edwards, 1992; Edwards and Baker, 1993; Von Caemmerer, 2013). In addition, CFA can solve the problem of inconvenient operation of leaf gas-exchange measurement in production, for example, the installation of leaf chambers (e.g. BERMONIS) and the inspection of their air tightness (the main obstacles of leaf gas exchange in commercial greenhouse production). While both methods are suitable to measure plant photosynthetic productivity (each with pros and cons), the cuvette based leaf gas exchange measuring method delivers direct measurement response, while CFA is an indirect procedure but with a faster response in some situations.

With the underlying physiological process of plant photosynthesis, CFA provides insights into the relationship between chloroplast electron transport rates and carbon metabolism. Some scholars reported that CFA parameters could be used to indirect predict *A_L_* by measuring the electron transport rateof PSII (*J_f_*) as under some conditions a linear relationship between *A_L_* and *J_f_* exists (Krall and Edwards, 1992; Herppich and Peckmann, 2000; Yin and Struik, 2009). In addition, quantum yield of PSII (Φ_2_) shows linear correlated with quantum yield of CO_2_ fixation (Edwards and Baker, 1993). These results are often obtained under favorable experimental conditions, e.g. when light radiation (*I*, µmol m^-2^ s^-1^) linearly increases during controlled light response curve measurements.

This study provides a valuable data set of photosynthetic physiological responses of plants in a dynamic production environment. Furthermore, it provides a method for estimating *A_L_* by using the chlorophyll fluorescence parameters, and provides an approach for maximizing photosynthesis by manipulating the environmental conditions with real-time detection of limiting factors of leaf photosynthesis in greenhouse environments.

## Materials and Methods

### Model Background

Around four decades ago a nowadays widely used biochemical photosynthesis model was proposed (Farquhar et al., 1980) (hereafter “FvCB model”). This model estimates *A_L_* as minimum of the Rubisco limited rate (*A_c_*, µmol CO_2_ m^-2^ s^-1^), the electron (e^-^) transport limited rate (*A_j_*, µmol CO_2_ m^-2^ s^-1^), and the triose phosphate utilization limited rate (*A_p_*, µmol CO_2_ m^-2^ s^-1^) of CO_2_ assimilation (Eqn. 1). Abbreviations for parameters are defined in **Table 1**.

(1) $$A_{L\;}=\text{min}(A_{c},\;A_{j},\;A_{p})$$

**Table 1:** Abbreviation used in this study.

Abb.	Definition	Unit
***A_L_***	Net photosynthesis rate	μmol CO_2_ m^-2^s^-1^
***A_c_***	Rubisco activity limited net photosynthesis rate	μmol CO_2_ m^-2^s^-1^
***A_gl_***	Gross leaf assimilation rate	μmol CO_2_ m^-2^s^-1^
***A_glmax_***	Maximum gross assimilation rate	μmol CO_2_ m^-2^s^-1^
***A_j_***	Electron transport limited net photosynthesis rate	μmol CO_2_ m^-2^s^-1^
***A_p_***	Triose phosphate utilization limited net photosynthesis rate	μmol CO_2_ m^-2^s^-1^
***C_a_***	Ambient air CO_2_ partial pressure or concentration	μbar
***C_c_***	Chloroplast CO_2_ partial pressure	μbar
***C_i_***	Intercellular CO_2_ partial pressure	μbar
***F***	Leaf chlorophyll fluorescence yield of light acclimated state	–
***Fm'***	maximal fluorescence yield of the light acclimated state	–
***f_OC_***	Ration of maximum oxygenation rate to maximum carboxylation rate	–
***f_cyc_***	A fraction of e- follows the cyclic mode around PS I	–
***f_pseudo_***	A fraction of e- follows the pseudocyclic mode for O_2_ reduction.	–
***g_b_***	Boundary layer conductance	mol m^-2^s^-1^
***g_m_***	Mesophyll diffusion conductance	mol m^-2^s^-1^
***g_s_***	Stomatal conductance	mol m^-2^s^-1^
*I*	Solar radiation	*μ*mol [photon] m^-2^s^-1^
***I_abs_***	Photon flux density absorbed by leaf photosynthetic pigments	*μ*mol [photon] m^-2^s^-1^
***I_inc_***	Photon flux density incident to leaves	*μ*mol [photon] m^-2^s^-1^
***J*_1_**	e- transport rate through PSI	*μ*mol [e^-^] m^-2^s^-1^
***J*_2_**	e- transport rate through PSI	*μ*mol [e^-^] m^-2^s^-1^
***J_f_***	Rate of e- transport calculated from the chlorophyll fluorescence measurement	*μ*mol [e^-^] m^-2^s^-1^
***J_NADP+_***	The electron transport for the NADP+ reduction	*μ*mol [e^-^] m^-2^s^-1^
***J_max_***	Maximum value of J under saturated light	*μ*mol [e^-^] m^-2^s^-1^
***K_C_***	Michaelis–Menten constant of Rubisco for CO_2_	μbar
***K_O_***	KmO Michaelis–Menten constant of Rubisco for O_2_	mbar
***M_CO_*_2_**	Molar mass of CO_2_	kg mol^-1^
***O***	Oxygen partial pressure	mbar
***P***	Pressure	Pa
***PAR***	Photosynthetically active radiation	*μ*mol m^-2^s^-1^
***P_i_***	inorganic phosphate	
***R***	Gas constant	J kg-1 K
***r_b_co_*_2_**	Boundary layer resistance to CO_2_ diffusion	s m^-1^
***r_C_co_*_2_**	Carboxylation resistance to CO_2_ diffusion	s m^-1^
***r_s_co_*_2_**	Stomatal resistance to CO_2_ diffusion	s m^-1^
***R_d_***	Day respiration (respiratory CO_2_ release other than by photorespiration)	μmol [CO_2_] m^-2^s^-1^
***T_a_***	Air temperature	K
***T_l_***	Leaf temperature	K
***V_Cmax_***	Maximum rate of Rubisco activity-limited carboxylation	μmol [CO_2_] m^-2^s^-1^
***VPD***	Vapour-pressure deficit between leaf and air	kPa
Г^*^	Cc-based CO_2_ compensation point in the absence of Rd	μbar
***θ_J_***	Factor for the degree of curvature	–
***ρ*_2_**	Proportion of Iabs partitioned to PSII	–
***α*_(_*_LL_*_)_**	Initial quantum yield	mol [e^-^] (mol photon)
***α_g_***	Nonreturned fraction of glycolate	μmol CO_2_ m^-2^s^-1^
**ϵ**	Light use efficiency by photorespiration	mg CO_2_J^-1^
***Φ_2_***	Quantum efficiency of PSII e- flow on PSII-absorbed light basis, usually assessed from the chlorophyll fluorescence measurements	–

The value of Rubisco limited rate (*A_c_*) is calculated as a function of the maximum carboxylation rate (*V_Cmax_*)

(2) $$A_{c}=\frac{（C_{c}-\Gamma^{*}）V_{Cmax}}{C_{c}+K_{mC}\left(1+O/K_{mO}\right)}-R_{d}$$

where *C_c_* is the CO_2_ partial pressure at the carboxylation sites of Rubisco, *K_mC_* and *K_mO_* are Michaelis-Menten constants of Rubisco for CO_2_ and O_2_, respectively (Farquhar et al., 1980). *R_d_* is the mitochondrial respiration of leaves.

According to the usage of energy suppliers NADPH and ATP, two similar equations with different parameter values (Eqns. 3 and 4) were used to estimate the RuBP-regeneration limitation, which is a function of the electron (e^-^) transport *J*:

(3) $$A_{j}=\frac{({\text{C}}_{\text{C}}-\Gamma_{*})J}{4{\text{C}}_{\text{C}}+8\Gamma^{*}}-R_{d}$$

(4) $$A_{j}=\frac{({\text{C}}_{\text{C}}-\Gamma_{*})J}{4.5{\text{C}}_{\text{C}}+10.5\Gamma^{*}}-R_{d}$$

Theoretically, *J* can be assessed by CFA (then *J* becomes *J_f_*). *J_f_* is given by:

(5) $$J_{f\;=\;}I_{inc}\cdot abs\cdot \rho_{2}\cdot {\text{Φ}}_{2}$$

where abs is the proportion of incident light that is absorbed by the leaf. It is frequently assumed to be 0.84 (Maxwell and Johnson, 2000) or 0.85 (Von Caemmerer, 2000); *ρ*_2_, is the fraction of absorbed light transported to PSII (frequently assumed to be 0.48; Von Caemmerer, 2000).

**Figure 1:**
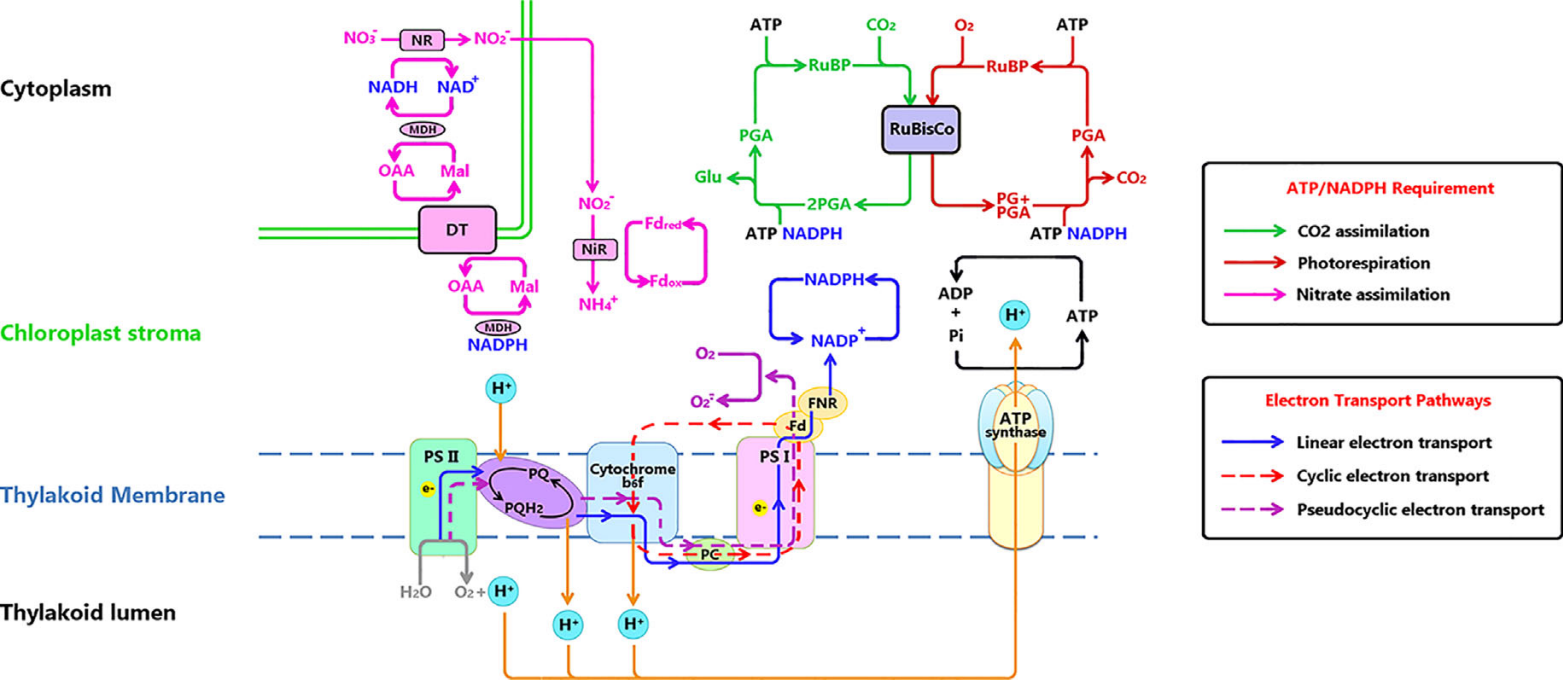
Electron transport chain on thylakoid membrane and related metabolisms. Three electron transport paths on thylakoid membrane: Linear electron transport (LET) (marked with blue line) is the electron (e^-^) flow transferred to NADP^+^, the end acceptor of LET for generating NADPH for CO_2_ reduction or photorespiration. Cyclic electron transport (CET) (marked with red dashed line) is the e^-^ flow transferred alone PSI, cytochrome b6f, plastocyanin back to PSI. Pseudocyclic electron transport (PET) (marked with purple dashed line) functions similar to LET, while the final e^-^ acceptor is O_2_. ATP and NADPH (originating from e^-^ transport), are used to drive CO_2_ assimilation in the Calvin Cycle (green line), photorespiration (dark red line) and NO_3_ assimilation (pink line). Notations: DT, Dicarboxylic acid transporter; Fd, Ferredoxin; FNR, Ferredoxin reduction system; Glu, glucose; RuBP, Mal, malic; MDH, Malate dehydrogenase; NIR, Nitrite reductase; NR, nitrate reductase; OAA, oxalocetate; PC, plastocyanin; PG, phosphoglycolate; PGA, phosphoglyceric acid; Pi, inorganic phosphate; PQ, plastoquinone; Ribulose-1,5-bisphosphate.

*J_f_* is assumed equal to the rate of e^-^ transport through PSII (*J*_2_), while *J*_2_ is the rate of e^-^ transport through PSI. The rate of cyclic e^-^ transport *J_cyc_* is *f_cyc_*·*J*_1_, where *f_cyc_* is a fraction of e^-^ follows the cyclic path (see **Figure 1**). This leads to the following balance as proposed by Yin et al. (2004).

(5-1) $$J_{2}+f_{cyc}\cdot J_{1}=J_{1}$$

We define all electron transport through PSI reaction center as 1, as well as the fractions e- for the cyclic and pseudocyclic paths (*f_cyc_* or *f_pseudo_*, respectively). The remaining fraction (1– *f_cyc_* – *f_psedo_*) is transferred to NADP^+^. The electron transport for NADP^+^ reduction $\left(J_{NADP^{+}}\right)$ can thus be formulated as:

(5-2) $$J_{NADP^{+}}=\left(1-f_{cyc}-f_{pseudo}\right)\cdot J_{1}$$

Combining Eqn. (5-1) and Eqn. (5-2), Eqn. (5-3) can be derived.

(5-3) $$J_{NADP^{+}}=\left(1-f_{cyc}-f_{pseudo}\right)\cdot \frac{J_{2}}{\left(1-f_{cyc}\right)}$$

We assume the environment is steady state, the pseudocyclic path, which may occur at high light condition to produce oxygen radicals $O_{2}^{\;-}$() is not considered, in this study (*f_pseudo_* = 0), means that:

(5-4) $$J_{NADP^{+}}=J_{2}=J_{f}$$

Finally, at high CO_2_ partial pressure (particularly in combination with high radiation) the rate of *A_L_* is sometimes limited by the release of inorganic phosphate (*P_i_*). Starch and sucrose synthesis may become inadequate to recycle the *P_i_* sequestered in the production of triose phosphates, in which case *P_i_* may become limiting (Woodrow and Berry, 1988; Harley and Sharkey, 1991; Lombardozzi et al., 2018). The *P_i_* limited part of *A_L_* (*A_p_*) is calculated as:

(6) $$A_{p}=\frac{3\;T_{p}(C_{C}-\Gamma_{*})}{C_{C}-\left(1\;+\;3\alpha_{g}\right)\Gamma^{*}}-R_{d}$$

Where *T_p_* is the triose-phosphate use (TPU) rate and *α_g_* is the non-returned fraction of glycolate.

In Eqns. 2 to 4 and Eqn. 6, the CO_2_ concentration on the chloroplast side (*C_c_*, µmol mol^-1^) is calculated from the pathway of ambient CO_2_ (*C_a_*, µmol mol^-1^) through leaf surface (*C_s_*, µmol mol^-1^) and intercellular air spaces (*C_i_*, µmol mol^-1^) to the chloroplast (Flexas et al., 2008; Flexas et al., 2012).

The leaf conductances to CO_2_, i.e. boundary layer conductance (*g_b_*, mmol CO_2_ m^-2^ s^-1^), stomatal conductance (*g_s_*, mmol CO_2_ m^-2^ s^-1^) and mesophyll conductance (*g_m_*, mmol CO_2_ m^-2^ s^-1^) are factors influencing *C_c_*. (Yin et al., 2009a) Due to the complicated leaf gas-exchange measurements for *g_m_* estimation, intercellular CO_2_ concentration is commonly assumed as: *C_i_* = *C_c_* (Farquhar et al., 1980; Ethier and Livingston, 2004; Manter and Kerrigan, 2004; Sun et al., 2014). However, as *g_m_* is a major variable in photosynthesis, neglecting *g_m_* will result in inaccurate prediction of *A_L_* (Nobel, 1977; Nobel, 1983; Warren, 2006; Pons et al., 2009; Yin and Struik, 2009; Yin et al., 2009a; Yin and Struik, 2012). The influence of this potential error in prediction *A_L_* has been considered and discussed in this study.

**Figure 2:**
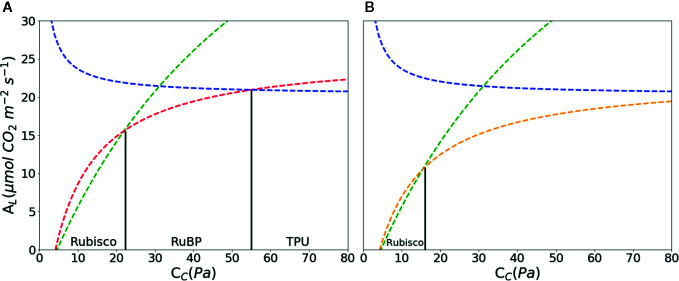
CO_2_ assimilation **(A)** as a function of chloroplast partial pressure of CO_2_ (*C_C_)* according to the FvCB model. With increasing *Cc*, *A_L_* is limited by ribulose-1, 5-bisphosphate (RuBP) carboxylase/oxygenase (Rubisco) marked with green, RuBP marked with red in A and orange in B; triose-phosphate utilization (TPU) marked with blue, respectively. Rubisco kinetics parameters were using values of **Table 1** in Sharkey et al. (2007): *K_C_* = 27.24 Pa, *K_O_* = 16.58 kPa, O = 21 kPa, Γ* = 3.74 Pa. Other parameters: *R_d_* = 1.0 μmol·m–2·s–1, V_Cmax_ = 97.0 μmol·m–2·s–1, *J_max_* = 144.0 μmol·m^–2^·s^–1^, *T_p_* = 7.1 μmol·m^–2^·s^–1^, *α_g_* = 0.15 μmol CO_2_ m^-2^s^-1^. Abbreviations for parameters are defined in **Table 1**. The RuBP limitation is calculated with Eqn. 3 **(A)** and Eqn. 4 **(B)**.

Two equations (Eqns. 3 and 4) were used to simulate electron transport limitation. The detailed derivation process is well described by Von Caemmerer (2000) and Yin et al. (2004). To simplify, in the production of NADPH and ATP, electron transport and the concomitant proton transfer in the chloroplast thylakoids are central processes. Carboxylation and oxygenation in C_3_ metabolic reactions requires NADPH and ATP. Farquhar proposed that each carboxylation requires 2 NADPH and 3 ATP, and each oxygenation requires 2 NADPH and 3.5 ATP. In Eqn. 3, the regeneration of RuBP is assumed restricted by NADPH, the rate of whole chain electron transport required to support NADPH consumption by the photosynthetic carbon reduction (PCR) and photorespiratory carbon oxidation (PCO) cycles during CO_2_ fixation (Von Caemmerer and Farquhar, 1981; Dubois et al., 2007; Sharkey et al., 2007). Therefore, oxygenation to carboxylation ratio is given by 2 Г^*^/C_i_ (Farquhar and von Caemmerer, 1982). The rate of NADPH consumption can be expressed as (2 + 4Г^*^/C_i_)*V*_c_, where *V*_c_ is the rate of carboxylation. Since the reduction of one NADP^+^ to NADPH requires two e^-^, the rate of e^-^ transport for satisfying the NADPH requirement is (4 + 8Г^*^/C_i_) *V*_c_ (**Figure 2**).

In Eqn. 4, the regeneration of RuBP is not only limited by NADPH, but also by ATP (Von Caemmerer and Farquhar, 1981; Bernacchi et al., 2003; Long and Bernacchi, 2003; Yin and Struik, 2009). The rate of ATP consumption in C_3_ reaction is (3 + 7Г^*^/C_i_) *V*_c_. The FvCB model assumes that 3 H^+^ are required for the photophosphorylation of 1 ADP to 1 ATP. Therefore, the flow of one e^-^ *via* the linear chain produces 2/3 ATP. Assuming ATP is produced by the linear e^-^ transport alone, the required rate of the linear e^-^ flow is (4.5 + 10.5Г^*^/C_i_) *V*_c_ (**Figure 2**) (Yin et al., 2004).

One key point of this study is the use of CFA to predict *A_L_*. Foyer and Noctor, 2002 Since the Mehler reaction (Mehler, 1951) is not subject to this study, we solely use the linear electron transport of steady state photosynthesis in Eqns. 3 and 4. ATP and NADPH are used to drive the CO_2_ assimilation, photorespiration and $NO_{3}^{-}$ assimilation (see **Figure 1**) (Robinson, 1987; Noctor and Foyer, 1998; Foyer and Noctor, 2002; Allen, 2003). In that, the assimilation of $NO_{3}^{-}$ has a lower requirement of ATP/NADPH, and furthermore, the reducing power for $NO_{3}^{-}$ assimilation may not directly originate from the chloroplast (Miflin, 1974; Yin et al., 2006; Walker et al., 2014). Meanwhile, whether the reductants and energy is come from linear electron transport is still unclear. The ferredoxin, NADPH, and ATP may partly be produced by cyclic or pseudocyclic electron flow. And the fraction for nitrate reduction is not a constant. It may depend on species, nutrient supply and growth stage (Yin et al., 2006). Due to the difficult endeavor of quantifying the proportion of electron flow for nitrate reduction in real time and the small proportion of it, the electron flow for $NO_{3}^{-}$ assimilation was not considered in this study.

### Plant Material, Growth Conditions

One hundred and forty-four tomato plants (cv. “Pureza”) were cultivated in February 2016 in a 62.6 m^2^ Venlo-type greenhouse at a commercial grower in Abtshagen, Germany (52°31'12.025'' N; 13°24'17.834'' E). The greenhouse had a side wall height of 4.2 m, equipped with double glass and single glass in the roof. The internal construction consisted of two double gutters in the middle and two single gutters beside the walls. Plants were planted in rock wools slabs with a common drip irrigation system. Seed was placed on rock-wall cubes on January 23rd 2016 and young plants were placed on the rock wools slabs two weeks after. Three weeks after transplanting, the measurements started. Temperature and humidity were controlled with pipe heating and passive roof ventilation. Set points for heating system were defined as 22 and 18°C for day and night, respectively; ventilation set point was 21°C day and night between April and October and 26°C for the rest of the year. The energy screen was unfolded one hour before sunrise and folded one hour after sunset. Between 7 a.m. and 8 p.m. supplementary light (high-pressure sodium lamps, HPSL) started when global radiations outside the greenhouse was below 20 W m^-2^. Water and nutrients were adequately supplied to the needs of the crop. Nutrient solution was adjusted with mineral fertilizer to an electric conductivity (EC) of 1.8 dS m^−1^ and a pH of 6.5. The nutrient concentration was used according to Lattauschke (2004) (**Table 2**).

**Table 2:** Nutrient concentration for greenhouse tomato used in this study.

Nutrient	Abr.	Amount	Unit	Nutrient	Abr.	Amount	Unit
**Nitrogen**	N	151	mg L^-1^	Iron	Fe	2.0	mg L^-1^
**Phosphorus**	P	37	mg L^-1^	Boron	B	0.3	mg L^-1^
**Potassium**	K	234	mg L^-1^	Copper	Cu	0.2	mg L^-1^
**Calcium**	Ca	128	mg L^-1^	Manganese	Mn	1.2	mg L^-1^
**Magnesium**	Mg	24	mg L^-1^	Molybdenum	Mo	0.05	μg L^-1^
**Sulphur**	S	110	mg L^-1^	Zinc	Zn	0.4	mg L^-1^

### Environment and Plant Photosynthesis Monitoring

The environmental variables air temperature, relative humidity, light, and CO_2_ concentration (*T_a_*, RH, *I*, and [CO_2_], respectively) were recorded by a commercial greenhouse monitoring system (Growwatch, Fytagoras BV, Leiden, The Netherlands). In this system, plant photosynthetic active radiation (PAR) was measured (Li-190R, LICOR, Lincoln, Nebraska, USA) as photosynthetically photon flux density (PPFD, µmol m^-2^ s^-1^). The monitoring system was placed on an uncovered area (right next to the plant) at the height of the seventh unfolded leaf (calculated from the top, the fifth leaf was usually the first mature leaf). *T_a_* and RH were measured by a commercial sensor for volume applications (HMP60, VAISALA, Helsinki, Finland). Leaf temperature (*T_l_*) of each seventh leaf of four plants was measured with an infrared radiation thermometer (CT11, HEITRONICS Infrarot Messtechnik, Wiesbaden, Germany). All variables were continuously measured and averaged over 5 min and stored on a central server. Outliers were detected according to Tukey (1977), i.e. an outlier is defined as a value that is smaller than the lower quartile minus 3 times the interquartile range, or larger than the upper quartile plus 3 times the interquartile range. Outliers and invalid measurements due to sensor calibration or failure were removed from the original data-set (Grubbs, 1950; Aggarwal and Yu, 2005). Scattered data outliers within PAR measurements as artifact based on sudden shade incidences hitting the PAR point-sensors during direct sunlight conditions (due to shade-spots of the greenhouse construction) were filtered with Savitzky-Golay filter (with order=3, window=21) (Orfanidis, 2006; Miranda, 2017); i.e., a mathematical procedure for smoothing data in order to increase data precision.

Leaf CO_2_ gas-exchange (GE) was recorded by the BERMONIS system, measuring the lump-sum of CO_2_ gas exchange of eight leaves and calculated to an averaged *A_L_*. Likewise, measurements of *T_l_*, the eight cuvettes were set at each seventh leaf of four plants. On each plant two opposing leaflets were used resulting in two cuvettes per plant. The fully expanded leaves were placed into the cuvettes (acting as transparent leaf chambers) with the leaves face-up, the metal frame supported the cuvettes in a horizontal position. A pilot experiment demonstrated the functionality of BERMONIS to commercial instruments (**Supplementary Material A**).

Leaf chlorophyll fluorescence yield of light acclimated state (F, -), and maximal fluorescence yield of the light acclimated state (F_m_′, -) were measured with a PAM monitor (Monitoring PAM, Walz, Effeltrich, Germany). The sensor was likewise BERMONIS set on the seventh leaves (on different plants). For that, F and F_m_′ were measured in the same time and quantum efficiency of PSII e^-^ flow on Φ_2_ of the leaf was calculated (Krause and Weis, 1984):

(7) Φ2=Fm'-FFm'

The sensors were re-placed to new leaves after two weeks. Recorded data are shown in **Supplementary Material B**.

### Model Development

**Figure 3:**
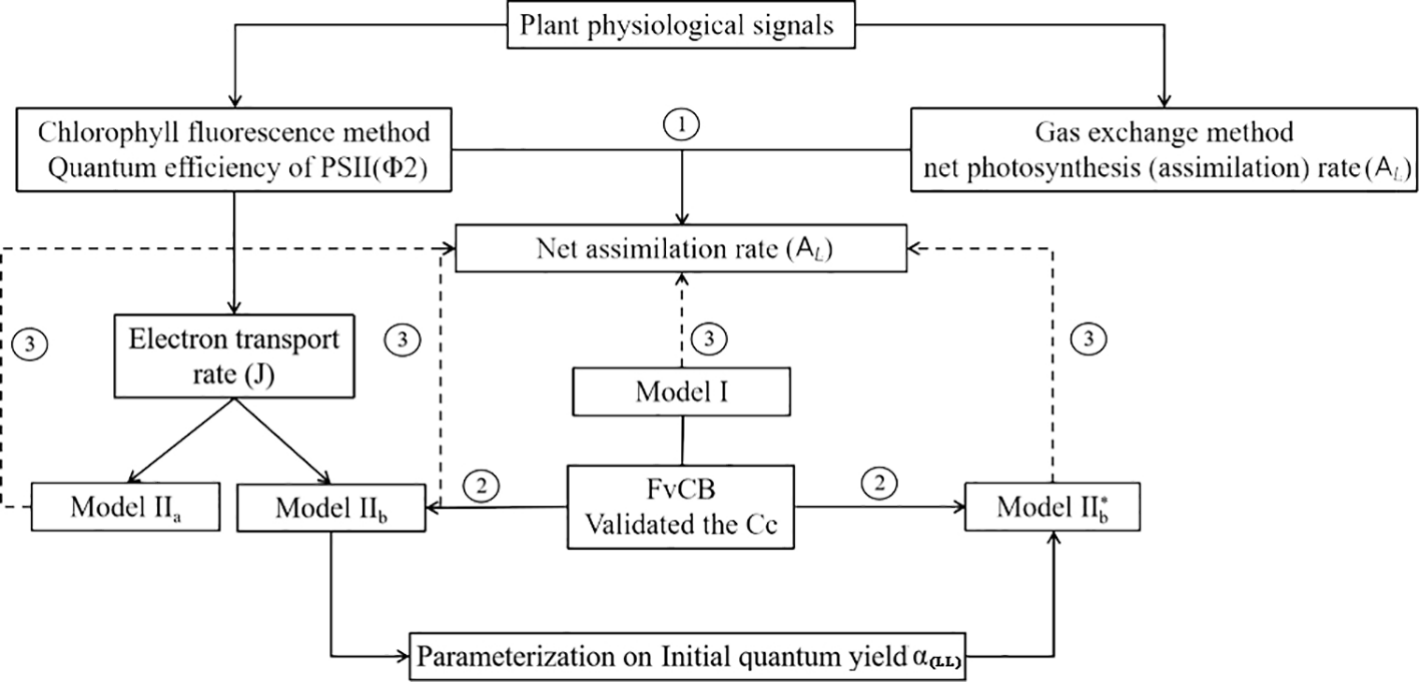
(1) Evaluation of two photosynthesis indicators with environmental factors; (2) Establishment of models: Model II_a_ and Model II_b_, a coupled model with common model (Model I) with FvCB model. In Model II_a_ *A_j_* is calculated with Eqn. (3), whereas in Model II_b_ *A_j_* is calculated with Eqn. (4), in both models *J* is calculated by measured Φ_2_. In Model II_b_* *J* is predicted with a non-rectangular hyperbolar curve Eqn. (10); (3) Evaluation of the three models with observed data.

A complete data set with environmental variables (*T_a_, T_l_*, RH, *I*, and [CO_2_]), mean *A_L_*, and Φ_2_ was established for model validation and parameters calibration in this study and structured as shown in **Figure 3**. Four models were compared in this study: Model I, Model II_a_ and Model II_b_, and Model II_b_* used in greenhouse leaf photosynthesis modelling (**Table 3**). Model I is the negative exponential light response commonly used in greenhouse leaf photosynthesis modelling (Thornley, 1976; Gijzen, 1992; Goudriaan and Van Laar, 1994; Körner et al., 2002; Körner, 2004). Model II_a_ and II_b_ use Model I as basis and further employ the complete FvCB model with two different equation to estimate *Aj*. In these models, *J* is estimated by detected Φ_2_ (Eqn. 5). In Model II_b_*, *J* is calculated with the non-rectangular hyperbolar curve (Eqn. 10).

**Table 3:** A summary of model formulas.

Model	Calculation equations
**Model I**	Eqn. (8), (9) & **Supplementary Material C**
**Model II_a_**	Use Model I and Eqn.(11),(12) to calculate *C_C_*, Eqn.(5) to simulate J, Eqn.(1),(2),(3),(6) to simulate *A_L_*
**Model II_b_**	Use Model I and Eqn. (11),(12) to calculate C_C_, Eqn.(5) to simulate J, Eqn.(1),(2),(4),(6) to simulate *A_L_*
Model II_b_*	Use Model I and Eqn. (11),(12) to calculate *C_C_*, Eqn.(10) to calculate J, Eqn.(1),(2),(4),(6) to simulate *A*_*L*_

#### Model I

In Model I, *A_L_* is determined from gross leaf assimilation rate (*A_gl_*, µmol CO_2_ m^-2^ s^-1^) minus leaf day respiration (*R_d_*, µmol CO_2_ m^-2^ s^-1^).

(8) $$A_{L}=\;A_{gl}-R_{d}$$

*A_gl_* is determined from light use efficiency ϵ and maximum gross assimilation rate *A_gl__max_* with the negative exponential light-response curve (Goudriaan and Van Laar, 1994) using absorbed radiation (*I_abs_*) as input.

(9) $$A_{gl}=\;A_{glmax}\cdot \left(1-e^{-\frac{\epsilon \;I_{abs}}{A_{glmax}}}\right)$$

This leaf photosynthesis light response model is commonly used to upscale leaf photosynthesis to the crop level (*A_crop_*) considering light distribution with Gaussian integration over the crop depth (Goudriaan and Van Laar, 1994). It is commonly used as basic photosynthesis model in many crop growth models for yield estimation (Spitters et al., 1989; Hoogenboom et al., 1990; Jones et al., 1991; Heuvelink, 1996). The description of the biochemical processes is simplified. The key parameters in this model could be identified by curve fitting or converted from *V_Cmax_* or *J_max_*. Detailed equations are shown in **Supplementary Material C**.

#### Model II_a_/II_b_

The steady-state version of the FvCB model has had the strongest impact and has become the standard model for photosynthesis of C_3_ species (Sharkey, 1984; Sharkey, 1985; Farquhar et al., 2001; Long and Bernacchi, 2003). The models predicts photosynthesis as the minimum of the *A_c_*, *A_j_*, *A_p_* (Eqn. 1, see Section Model background). In both Models II_a_ and II_b_, *J* is assessed by chlorophyll fluorescence *J_f_*. While Model II_a_ includes Eqn. 3, Model II_b_ is using Eqn. 4 for *A_j_* simulation.

#### Model II_b_*

In Model II_b_*, *J* is calculated with a non-rectangular hyperbolic curve of incident light (Farquhar and Wong, 1984). Solely environmental variables were used for parameter fitting of Model II_b_*.

(10) $$J=\;\frac{J_{max}+\alpha_{\left(LL\right)}\cdot I_{abs}-\sqrt{{\left(J_{max}+\alpha_{\left(LL\right)}\cdot I_{abs}\right)}^{2}-4\theta_{J}\cdot J_{max}\cdot \alpha_{\left(LL\right)}\cdot I_{abs}}}{2\theta_{J}}$$

where *I_abs_* µmol [ photon ] m^−2^s^−1^ is the absorbed light; *α_(LL)_* is a factor covert absorbed light to the useful light absorbed by PSII. Therefore, *α_(LL)_*·*I_abs_* represents light absorbed by PSII. *θ_J_* is a factor for the degree of curvature, assumed as 0.85 (Ögren and Evans, 1993; Von Caemmerer, 2000; Ethier et al., 2006).

#### Estimating Chloroplast CO_2_ Partial Pressure

*C_C_* is derived from the pathway of CO_2_ from ambient *C_a_* through leaf surface *C_s_* and intercellular air spaces (*C_i_*). Here, *g_b_*, *g_s_* and *g_m_* are indicated (Flexas et al., 2008). We analyzed the predicted results with and without calculated *g_m_.* According to Fick's first law of diffusion for CO_2_ transfer along the path from *C_a_* to *C_c_* is given by:

(11) $$C_{C}=C_{i}-A_{L}\left(\frac{1}{g_{m}}\right)$$

(12) $$C_{i}=C_{a}-A_{L}\left(\frac{1}{g_{b}}+\frac{1}{g_{s}}\right)$$

As shown in Eqns. 11 and 12, *A_L_* is required to be known a priori. To avoid infinite circulation of variables, the initial estimated *A_L_* rate of Model I is used as starting point for *C_c_* and *C_i_* calculation.

In some calculations, *C_i_* is treated equal to *C_c_* (Leuning, 2010) and it was suggested setting *g_m_* to be finite large (*g*_*m*_→*∞*) (Björkman, 1973; Laisk et al., 2005). Then Eqn. 11 can be re-formulated to Eqn. 12 and thus the need for *g_m_* will be redundant:

(13) $$C_{C}≅C_{i}=C_{a}-A\left(\frac{1}{g_{b}}+\frac{1}{g_{s}}\right)\;\;\;\;\;\;\;\;\;$$

We propose the Jarvis model as sub-model for *g_b_* and *g_s_*.

(14) $$g_{b}=\frac{P}{R\cdot T\cdot r_{b\_co2}}$$

(15) $$g_{s}=\frac{P}{R\cdot T\cdot r_{s\_co2}}$$

Where P/RT is the coefficient used to convert the resistance units (s m^-1^) to molar units. *r_b_CO_*_2_ and *r_s_CO_*_2_ are the boundary-layer resistance and stomatal resistance respectively (**Supplementary Material C**).

Goudriaan and Van Laar (1994) suggested an equation for calculating carboxylation resistance (*r_C___CO_*_2_). In theory, *r_C___CO_*_2_ can be used to calculate *g_m_* as:

(16) $$g_{m}=\frac{P}{R\cdot T\cdot r_{C\_co2}}$$

Consequently, the CO_2_ partial pressure within the chloroplast was calculated with and without inclusion of *g_m_* and the respective results were compared. In this context *C_C_* was calculated as:

1. Without *g_m_*, assuming that *g*_*m*_→*∞*; (See Eqn. 13)
2. With *g_m_* based on the inverse *r_C_CO_*_2_, namely *g*_*m*_=*f*(*r*_*C*_*co*2_) (*r*_*C*_*co*2_ see **Supplementary Material C**)
3. With a hypothetical value, from the perspective of optimal fitting of the model, set *g_m_* as 0.25 mol m^-2^ s^-1^. This value (*g_m_* = 0.25 mol m^-2^ s^-1^) is consistent with an average mesophyll conductance of annuals herbaceous (Flexas et al., 2008; Yin et al., 2009b).

#### Estimating the Rate of Photosynthetic Electron Transport

The value of *α_(LL)_* differs among published literature in e.g. Von Caemmerer, 2000; Niinemets et al. (2009), or Yin et al. (2009b). Three values of *α_(LL)_* were compared: two values were reported in literatures (Niinemets et al., 2009; Yin et al., 2009b); one value was estimated with the parameter optimization method in this study (*α_(LL)_* = 0.405).

Curve fitting was used for parameter estimation of *g_m_* and *α_(LL)_*. The nonlinear least squares procedure available in python scipy.optimize tool box (function leastsq) was applied to minimize the sum of the residuals between measured data and predicted data (Madsen et al., 2004; Wallach et al., 2006; Salazar-Moreno et al., 2017).

### Model-Parameterization

The biochemical parameters *V_Cmax_* (μmol m^-2^ s^-1^) and *J_max_* (μmol m^-2^ s^-1^) were assessed with an open leaf gas exchange measuring system (LI-6400, Li-Cor Inc., Lincoln, Nebraska, USA). The system was used to create CO_2_-response curves (commonly referred to as A-Ci curve) at a CO_2_ concentration set at a course of different set points (i.e. 400, 350, 300, 250, 200, 100, 400, 450, 500, 550, 600, 800, 1,000 μmol mol^-1^), while keeping PAR at 1,500 μmol m^-2^ s^-1^ PPFD. Measurements were made at pre-set leaf temperature set points of 25°C and the system was set such that each CO_2_ level was reached constant for several seconds and the measurement was recorded at this point. Data of the three measurements were averaged for further calculations. The *A-Ci* curve fitting was carried out using the Ethier and Livingston method (Ethier and Livingston, 2004; Ethier et al., 2006).

### Statistical Analysis and Model Performance

For all statistical analyses, the statistical software package SPSS was used (version 23.0, UNICOM Global, CA, USA). Multiple linear regression (MLR) was used for examining *A* and Φ_2_ response to multi-environmental variables: air temperature (*T_a_*, K), vapour pressure deficit (VPD, kPa) and PAR. The coefficient of determination (R^2^), root mean squared error (RMSE), and mean absolute error (MAE) were used to analyze the goodness-of-fit between the simulated value and the measured value. RMSE and MAE were used to evaluate the model performance. The smaller the RMSE and MAE value, the better the consistency between the simulated and the measured value, thus the more accurate and reliable the model prediction becomes (Chai and Draxler, 2014).

## Results

### Evaluation of Physiological Signals

Photosynthetic signals are indicators of plant health and can be used as variables to formulate control strategies, when compared with the expected optimum at current environmental conditions. For multi linear regression, the collinearity of factors needs to be taken into account. These environmental factors meet the collinearity diagnostics with the variance inflation factor less than 10 (data not shown). The resulting regression equations are presented in **Table 4**. The results showed that the environmental variables can better explain the variation of *A_L_* than the variation in Φ_2_: 61.5% of the variation of *A_L_* could be assessed by *T_a_*, VPD, and *I*, whereas only 50.2% of Φ_2_ variation can be assessed by environmental factors Thus, in our measurements *A_L_* is a better suited to evaluate plant responses to the environmental factors than Φ_2_.

**Table 4:** Multiple linear regression: the environmental variables explain variance in *A_L_* andΦ_2_.

Ind.	Ta	VPD	PAR	Regression equation	R^2^
***A_L_***	69.73	11.01	19.26	y=-12.7+0.89Ta-1.43 VPD +0.002 PAR	0.615
**Φ_2_**	79.90	17.18	3.34	y=1.1-0.02Ta+0.04 VPD +6.168×10-6 PAR	0.502

### Model Validation and Parameters Calibration

**Figure 4:**
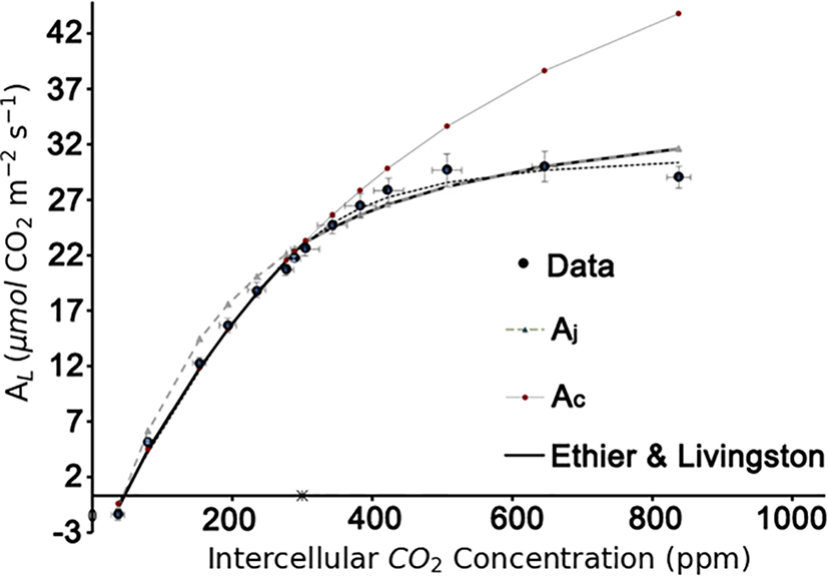
Estimation of the biochemical parameters by A-Ci curve fitting based on the Ethier-Livingston method. Data points represent the mean value of three leaf replicates.

In the present study, the method proposed by Ethier and Livingston (2004) was used to identify the biochemical parameters *V_Cmax_*_,_ *J_max_*_,_ *R_d_*. With a well-fitting result (R^2^ = 0.99; **Figure 4**). *V_Cmax_*_25_ = 71.0 μmol m^-2^ s^-1^, *J_max_*_25_ =147.7 μmol m^-2^ s^-1^, and *R_d_*_25_ = -0.34 μmol m^-2^ s^-1^ were used in the further modelling framework.

**Figure 5:**
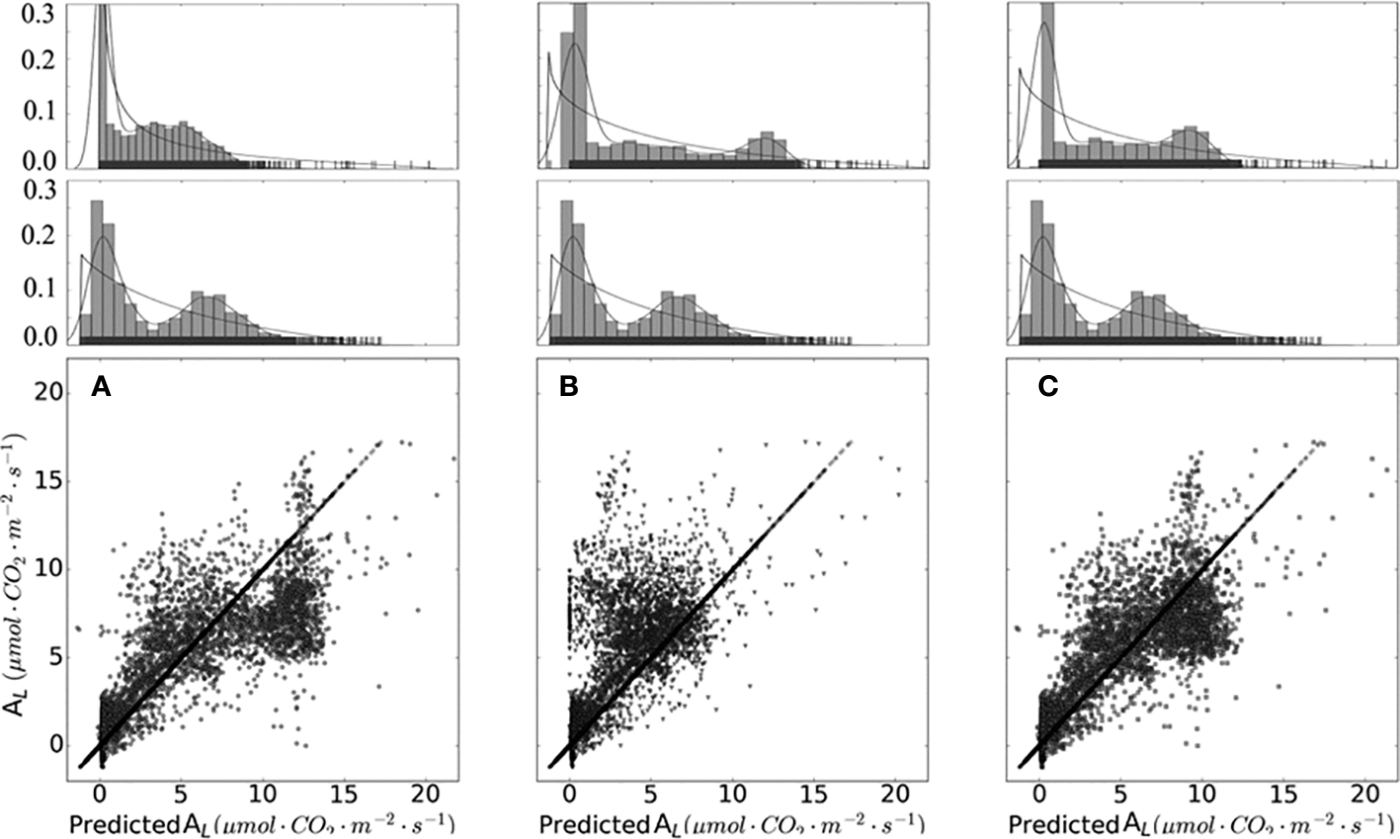
Measured and simulated net photosynthesis rate based on different chloroplast CO_2_ partial pressure. Data points shown in **(A)** were calculated with infinity mesophyll conductance (*g_m_*). Data points shown in **(B)** were calculated based on converted *r_C_co2_*. Data points shown in **(C)** were calculated with calibrated *g_m_* equal to 0.25. On the top of **(A–C)** are sub-graphs, displaying the data distribution of each plot, on the middle layer of **(A–C)** displayed the distribution of measured data.

From **Figure 5** and **Table 5**, it is evident that calibrated *g_m_* improved the prediction quality with the highest coefficient of determination (R^2^), and achieved the lowest RMSE. Considering *g_m_* infinite, the simulation results are overestimated. *g_m_*, based on optimal fitting, equal to 0.25, achieved the highest R^2^ and the lowest RMSE and MAE.

**Table 5:** Coefficient of determination (R^2^), root mean square of error (RMSE) and mean absolute error (MAE) of the linear regression calculated between the observations and simulations of mesophyll conductance g_m_.

No.	*g_m_*	R2	RMSE	MAE
**1**	*g*_*m*_→*∞*	0.57	2.39	1.61
**2**	*g_m_* = f(r_C_)	0.52	2.56	1.61
**3**	*g_m_* = 0.25	0.71	1.99	1.34

**Figure 6:**
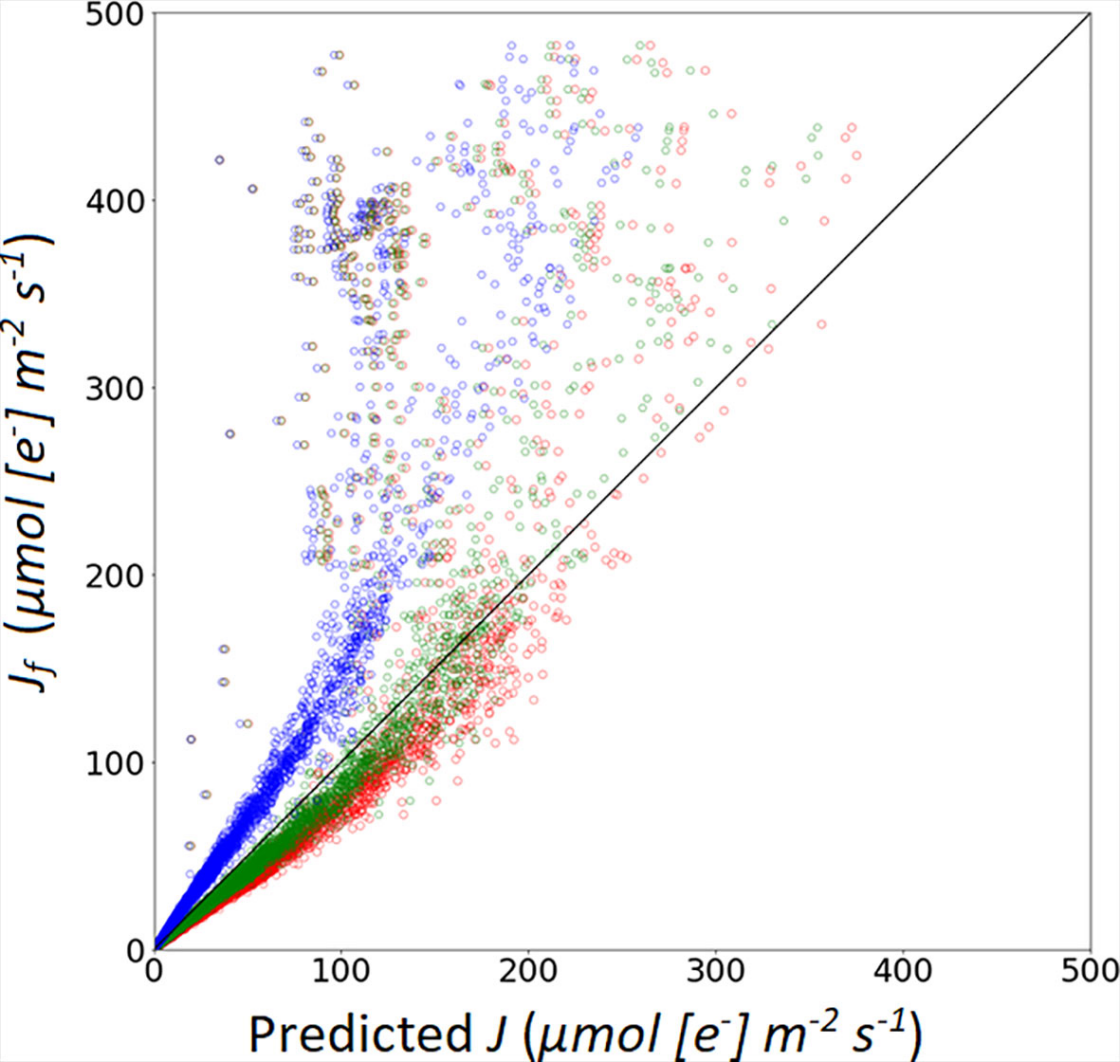
Simulation of photosynthetic electron transport rate based on: A recommended value of α from Niinemets et al. (2009), marked with red open circles; a recommended α value from Yin et al. (2009b), marked with blue open circles; a calibrated α value (0.405), marked with green open circles.

Eqns. 3 and 4 are two approaches to calculate *A_j_* (**Table 7**). In Model II_b_ (using Eqn. 4) yielded a higher R^2^ and lower RMSE compared to calculations with Model II_a_ (i.e. using Eqn. 3). Using Eqn. 10 to simulate the measured *J_f_*, *α*_(_ *_LL_*_)_ value impacted the prediction performance of the model. Three *α*_(_ *_LL_*_)_ values were used in this study. From **Figure 6**, the results show that the calibrated parameters can largely improve the prediction quality (**Table 6**). RMSE and MAE decreased after applied the calibrated *α*_(_ *_LL_*_)_ value, which indicated that the R^2^ and RMSE between predicted value and measured value decreases after changing *α*_(_ *_LL_*_)_ and was used for Model II_b_*.

**Table 6:** Coefficient of determination (R^2^), root mean square of error (RMSE) and mean absolute error (MAE) of the linear regression calculated between the observations and simulations of electron transport rate *J* calculated with the three conversion factors (***α***_(_ *_LL_*_)_) values.

Marked color	*α*_(_ *_LL_*_)_ value	reference	R^2^	RMSE	MAE
**blue**	0.24	Yin et al., 2009b	0.85	52.76	18.4
**green**	0.40	Optimized	0.74	47.35	13.58
**red**	0.46	Niinemets et al. (2009)	0.70	47.76	16.08

**Table 7:** Coefficient of determination (R^2^), root mean square of error (RMSE) of the linear regression calculated between the observations and Models.

Model	R^2^	RMSE	MAE
**Model I**	0.64	2.21	1.50
**Model II_a_**	0.70	1.99	1.34
**Model II_b_**	0.71	1.98	1.33
Model II_b_*	0.71	1.99	1.34

### Model Test

**Figure 7:**
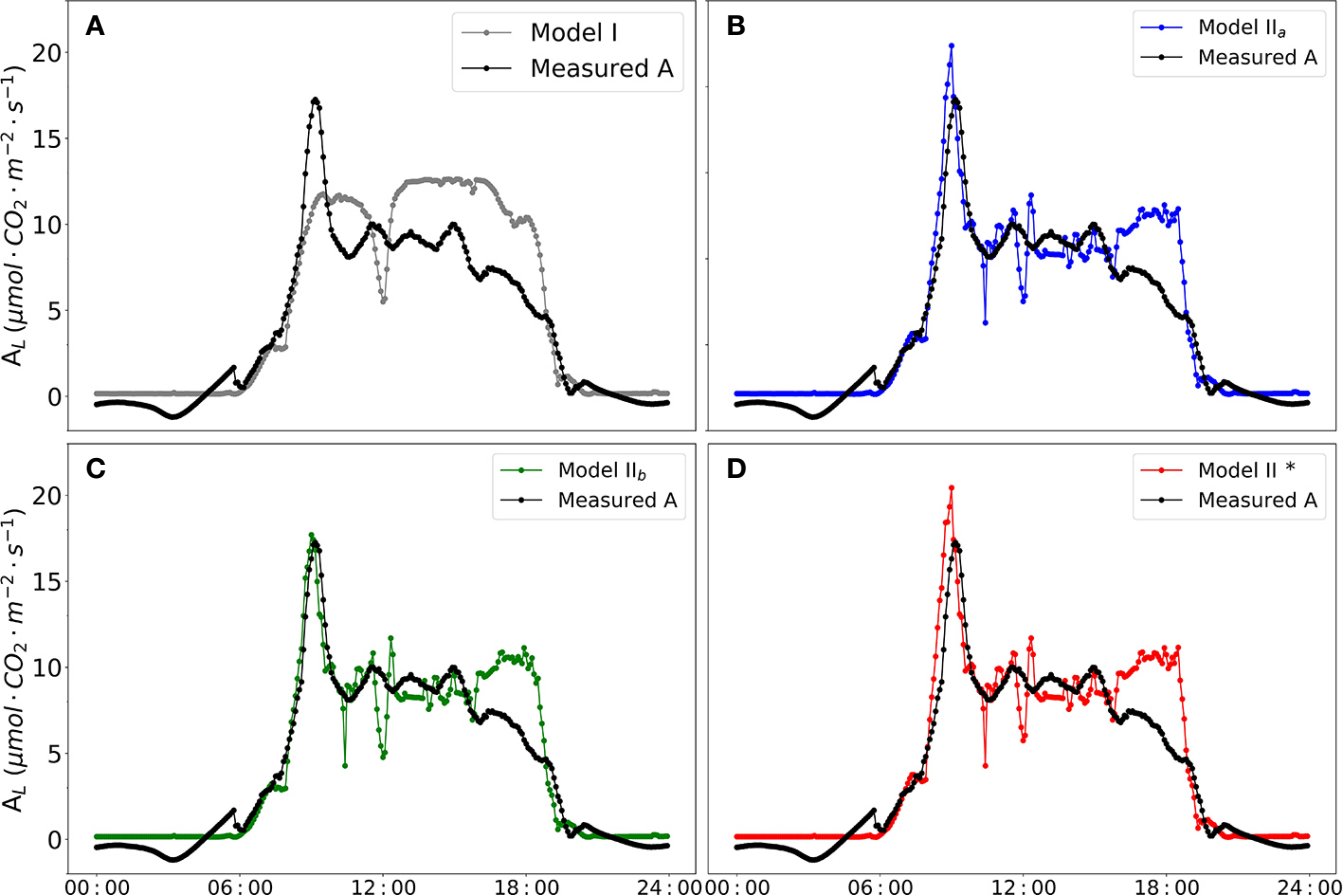
Diurnal dynamics of net photosynthesis rate. Data points shown in black circles are actual value measured by BERMONIS **(A–D)**. Data points shown in filled gray circles in **(A)** are net photosynthesis rate calculated with Model I. Data points shown in filled blue circles in **(B)** are calculated with Model II_a_. Data points shown in filled green circles in **(C)** are calculated with Model II_b_. Data points shown in filled red circles in **(D)** are calculated with Model II_b_*. V_Cmax_ = 71.0 μmol CO_2_ m^-2^ s^-1^, J_max_ = 147.7 μmol [e^-^] m^-2^ s^-1^, R_d_ = -0.34 μmol CO_2_ m^-2^ s^-1^, *g_m_* = 0.25 mol m^-2^ s^-1^ were applied in the Model II_a_, Model II_b_ and Model II_b_*. In Model II_b_* *α_(LL)_* =0.405 was applied in the modelling framework.

The diurnal changes of the net photosynthesis rate and three models were illustrated in **Figure 7**. In contrast to Model I, the three versions of Model II (Model II_a_, Model II_b_ and Model II_b_*) improved the prediction of *A_L_* (i.e. higher R^2^, **Table 7**). Implementing the predictions obtained from CFA into the model family Model II (i.e. with Model II_b_) yielded in a high R^2^ of 0.71. In addition, parameterization of *α*_(_ *_LL_*_)_ as part of calculation of *J* (Eqn. 10) could be used to well predict *A_L_*.

### Model Application

**Figure 8:**
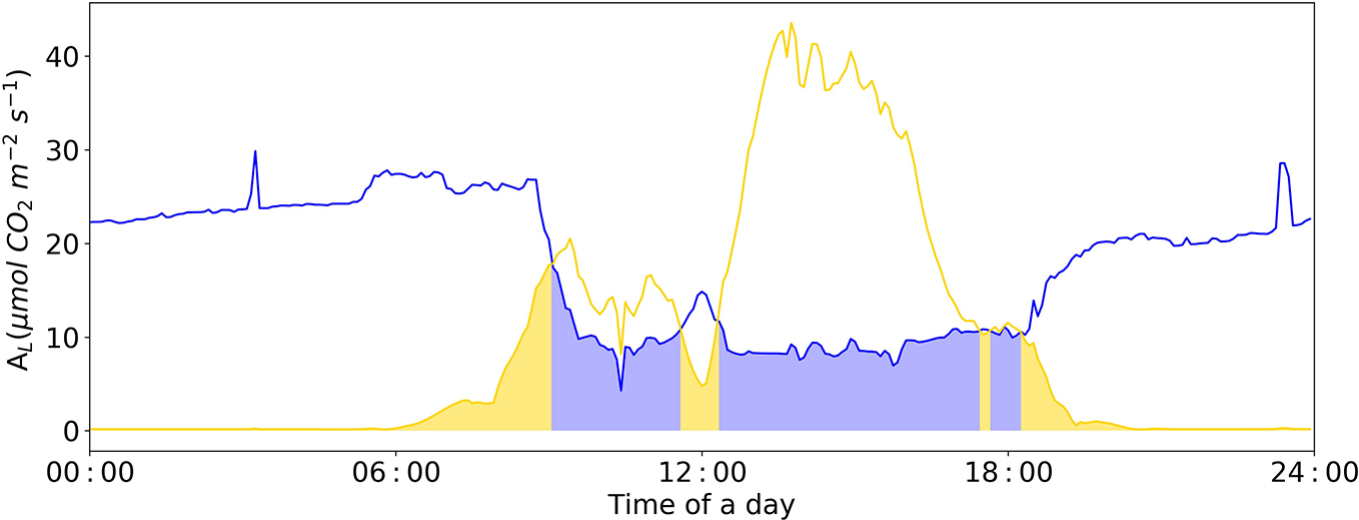
Diurnal dynamics of net photosynthesis rate for Rubisco and electron-transport limited rates calculated with Model II_b_. The Blue line represents Rubisco carboxylation-limited assimilation rate (*A_c_*). The yellow line represents electron transport-limited assimilation rate (*A_j_*). The yellow area represents leaf assimilation limited by *A_j_*; the blue area represents leaf assimilation limited by *A_c_*. *V_Cmax_* = 71.0 μmol CO_2_ m^-2^ s^-1^, *J_max_* = 147.7 μmol e^-^ m^-2^ s^-1^, *R_d_* = -0.34 μmol CO_2_ m^-2^ s-^1^, *g_m_* = 0.25 mol m^-2^ s^-1^ were applied in the Model II_b_.

During night, the ambient CO_2_ concentration in the greenhouse increases due to plant respiration (**Figure 8**). At this time, the limiting factor of photosynthesis is the insufficient electron transfer efficiency of chloroplasts caused by insufficient light. During the light period, photosynthesis consumes ambient CO_2_, and without CO_2_-supply its concentration in the greenhouse air rapidly decreases. Under this condition, the limiting factor of photosynthesis is shifted to “Rubisco-limitation”, a close relation to ambient CO_2_ concentration (**Figure 8**).

**Figure 9:**
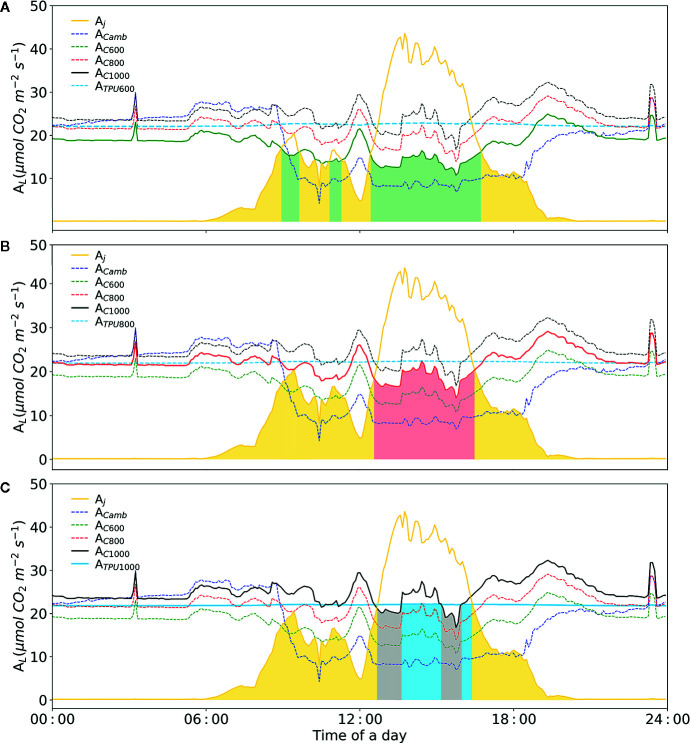
Diurnal dynamics of net photosynthesis rate for electron-transport and carboxylation limited rates calculated with Model II_b_. **(A)** Simulation of diurnal net photosynthesis rate with air CO_2_ concentration of 600 μmol mol^-1^. **(B)** Simulation of diurnal net photosynthesis rate with air CO_2_ concentration of 800 μmol mol^-1^. **(C)** Simulation of diurnal net photosynthesis rate with air CO_2_ concentration of 1000 μmol mol^-1^. The yellow line represents electron transport-limited assimilation rate (A_j_). The blue dashed-line represents rubisco carboxylation-limited assimilation rate (A_c_) at the given CO_2_ concentration. The sky-blue line represents the TPU limited assimilation with corresponding CO_2_ concentration. The three colors, green, red and black represent simulations with three CO_2_ concentrations, i.e. 600, 800, 1000 μmol mol^-1^, respectively. The colored area represents leaf assimilation rate. *V_Cmax_* = 71.0 mmol CO_2_ m^-2^ s^-1^, *J_max_* = 147.7 mmol e- m^-2^ s^-1^, Rd = -0.34 mmol CO_2_ m^-2^ s^-1^, gm = 0.25 mol m^-2^ s^-1^ were applied.

Based on our designed soft-sensor system, the CO_2_ concentration required by plants in the current cultivation environment can be calculated accurately. For instance, as illustrated in **Figure 9** (before 01:00 p.m.), supplying excessive CO_2_ to concentrations of 1,000 μmol mol^-1^ under insufficient lighting conditions does not improve the rate of photosynthesis, resulting in the waste of CO_2_ (Schmidt, 1998). Meanwhile, when dosing extra CO_2_, duo to the limited inorganic phosphate (*P_i_*) TPU limitation is likely to occur. The soft-sensor can improve the understanding and control of plant photosynthesis, so as to potentially improve greenhouse climate control.

## Discussion

Net photosynthesis prediction in a tomato crop can be improved significant when on-line measurements with sensor systems and intelligent algorithms of models are combined to a so-called soft-sensor (De Koning, 2006; Körner, 2019). Here, the combination of real-time chlorophyll fluorescence measurements and photosynthesis models is suggested. However, when model-predicted rates of CO_2_ exchange are compared with measured gas exchange, measuring accuracy of a gas exchange measurement system may complicate the determination of the true net photosynthesis. In this study, with the BERMONIS (Schmidt, 1998; Schmidt, 2005) a well-tested multi-leaf cuvette system was used for gas exchange measurements (Huber, 2011; Dannehl et al., 2014).

Another problem in designing soft-sensors often lies in the model structures. Here the difficulty is the unsuitability of the models for direct usage in a soft-sensor. The prediction efficiency of the used models depend among others on the identification and the estimation of substrate concentration, the chloroplast CO_2_ partial pressure (*C_C_*), and the photosynthetic electron transport rate (*J*). While (*C_C_*) can be estimated from the calculated intercellular CO_2_ concentration (*C_i_*), *A_L_* and *g_m_* needs to be known beforehand. However, in general, *C_i_* and *A_L_* are also unknown at the beginning and a consequential model nesting tends to get trapped in infinite loop in simulations. Therefore, the key issue of using the FvCB models to calculating the actual photosynthetic rate is to accurately provide *C_C_* and *J* data, either attained through model calculations or by measurements. To solve this problem, a commonly used but simplified biochemical *A_L_*-model with negative exponential light-response (Model I) was coupled with the full biochemical model approach based on Farquhar et al. (1980) for calculating *C_C_*, for which, in turn, *g_m_* needed to be identified. Niinemets et al. (2009) and Flexas et al. (2012) evaluated the importance of *g_m_* in estimation of net photosynthesis rate. It was demonstrated that a hypothetical situation, with *g*_*m*_→*∞*, which means there is no diffusion restriction in the mesophyll, resulted in higher daily photosynthesis, than any other parameterization. This is consistent with the conclusion of this study. Due to the assumption of a finite *g_m_*, a resistance between intercellular air spaces and the Rubisco carboxylation-sites in chloroplasts was used (Farquhar and Wong, 1984; Flexas et al., 2008). Results show that this equation does not fit *A_L_* very well. However, at daytime, the lower *g_m_* value resulted in lower *C_C_* values and led to the underestimation of *A_L_*. Therefore, we recommend a “universal” or cultivar dependent correction factor, or the usage of estimating *g_m_* according to different experiments.

For *A_L_* model estimations with Model I (used by Körner, 2004), maximum carboxylation rate and maximum electron transport rate (*V_Cmax_* and *J_max_*) need to be known. A general model for calculation of *V_Cmax_* and *J_max_* was reported by Farquhar et al. (1980). In this model, *V_Cmax_*_25_ (i.e. *V_Cmax_* at 25°C) is calculated with superficial chlorophyll density (ρ_chl_; assumed as 0.45 g m^-2^), the turnover number of RuBP (carboxylase) (*k_C_*; assumed as 2.5 s^-1^), and the total CO_2_ concentration of enzyme sites (*E_t_*; assumed as 87.0 μmol g^-1^. *J_max_*_25_ was computed as 467 times ρ_chl_ (Van Ooteghem, 2007). The calculated results of *V_Cmax_*_25_ and *J_max_*_25_ were 97.875 and 210.15 μmol m^-2^ s^-1^, which are different from our predictions. These values lead to an overestimation of *A_L_* (Model I). This underlines the insecure prediction quality of Model I and the central importance of carboxylation rate and maximum electron transport rate in photosynthesis prediction models.

For light-limited assimilation (*A_j_*, electron transport-limited rate of photosynthesis), there are two widely used forms of the equations, i.e. Eqns. 3 and 4. The rate of carboxylation when electron transport is limiting has not yet been described unambiguously in the FvCB model. As discussed by Yin et al. (2009b), for Eqn. 3, RuBP regeneration is assumed limited because of insufficient NADPH. Von Caemmerer (2000) elaborated that Eqn. 4 assumes ATP limiting: its two forms result from different assumptions about the operation of the Q cycle and the number of protons (H^+^) required for synthesizing an ATP. Yin et al. (2009b) proposed that the stoichiometric relationship in Eqn. 4 assumes linear electron transport limited by ATP. Our results show a higher R^2^ and lower RMSE with Eqn. 4, implying that in common production condition RuBP regeneration is likely limited by ATP. It can be deduced that under actual production conditions, ATP deficiency may be more related to RuBP regeneration limitation. (Qian et al., 2012), As there are some unknown pathways that cannot be fully represented by this model, the reverse cannot be supported by our data. The model discussed in this study, mainly concerns the basic circumstance (steady-state). A more robust model in unstable conditions could be the model proposed by Yin et al. (2004). However, with this model, constraints for the dynamic variables are needed.

Furthermore, when using Eqn. 10 to calculate the electron transport rate, the essential parameter is *α*_(_ *_LL_*_)_. *α*_(_ *_LL_*_)_ can be gained by mathematical curve fitting. Therefore, once *α*_(_ *_LL_*_)_ is known, Eqn. 10 can be used to estimate the electron transfer rate in the absence of CFA.

In our model analysis, we have used real greenhouse data, in order to clearly interpret that a soft-sensor system can provide accurate information. In our simulations we used *ceteris paribus* conditions, as only one variable (CO_2_ concentration) was changed, while the other potentially dynamic parameters were set fixed. However, as *V_Cmax_* is an exponential function of light (Von Caemmerer and Edmondson, 1986; Brooks et al., 1988; Arulanantham et al., 1990; Makino et al., 1994; Qian et al., 2012; Qian et al., 2015), it should be emphasized that light condition need to be considered in the practical application. The next step would therefore include a full simulation study (including sensitivity analysis) varying all external variables. Due to the difficult parameterization process of $NO_{3}^{-}$ reduction and its small contribution, the fraction of NO_3_-used by ATP and NADPH was not considered in this study. However, in future investigations, this could be explored in hydroponics nutrient composition experiments variating $NO_{3}^{-}$ or $NH_{4}^{+}$. This, nevertheless, was out of scope for the present research.

Our results suggest that (1) the CFA parameter Φ_2_ can be used to predict net photosynthesis rate and that (2) a parameterized photosynthetic electron transport rate model is suitable to predict measured electron transport rate and leaf photosynthesis. The combination of CFA measurements and mathematical modelling can be used for plant performance monitoring and furthermore used as a module for a DSS. The model performance expressed as R^2^ or RMSE was significantly improved with *J_f_*.

Up-scaling *A_L_* to *A_crop_*, i.e. from leaf to canopy photosynthesis, will include the heterogeneity of vertical leaf differences in age and light adaptation resulting in leaf morphological differences (e.g. Laisk et al., 2005). For that it is necessary to estimate the model parameters in *A_L_* -predictions with different vertical distribution in the canopy.

To summarize, in the present paper the basis of a monitoring system was introduced, which combines chlorophyll fluorescence analysis and model predictions using a biochemical leaf photosynthesis model (Model II_b_). The performance of a predictive model may be improved by combining it with the sensor-based on-line measurement of plant physiological parameters. The approach evaluated in this study provides information on the relationship between rates of photosynthetic electron transport and carbon gain. Furthermore, it could be used as the scientific basis for practical application of CO_2_ enrichment in the greenhouse. The next step will be the incorporation of morphological differences of leaves in a canopy to the proposed soft-sensor system.

## Conclusion

In summary, a soft-sensor, based on both sensors and models, is suitable to predicting rates of photosynthesis at the leaf level. However, for a well-fitting model system, a parameters validation of the biochemical parameters is needed. For estimating the CO_2_ concentration in chloroplasts, coupling of the Jarvis model with Model I can avoid a vicious cycle of parameters. Model II_b_ could reduce the effects of the errors of the simplified model as indicated by the reduced R^2^ between predicted data and measured data and the increased RMSE. Consequently, using these models as sub-systems in the soft-sensor approach could be a precise method for developing a greenhouse control strategy based on the direct evaluation of plant responses. However, differences in leaf morphology, which could result in different *V_Cmax_* and *J_max_* need to be exactly parameterized for a well performing DSS.

## Data Availability Statement

All datasets generated for this study are included in the article/**Supplementary Material**.

## Author Contributions

WY performed the measurements. US was involved in planning and supervised the work. WY and OK processed the experimental data, performed the analysis, drafted the manuscript, and designed the figures. All authors discussed the results and commented on the manuscript.

## Funding

We are grateful for the generous financial support received from Humboldt University of Berlin and the program of China Scholarships Council.

## Conflict of Interest

The authors declare that the research was conducted in the absence of any commercial or financial relationships that could be construed as a potential conflict of interest.
