# Supplementary material for: Crop Photosynthetic Performance Monitoring Based on a Combined System of Measured and Modelled Chloroplast Electron Transport Rate in Greenhouse Tomato
Source: Front Plant Sci. 2020 Jul 10;11:1038. doi: 10.3389/fpls.2020.01038 (PMC7381275; doi:10.3389/fpls.2020.01038)
Supplement: Supplementary file 1 [file DataSheet_1.docx]

**Supplementary Material A:**

**Comparison of laboratory and process gas exchange analyzers and evaluation of the consistency of measurement results with the Bland-Altman method (Comparison of BERMONIS and GFS-3000 gas exchange analyzers on diurnal measurements)**

Fig. S-1 shows the comparison of photosynthesis rate measurement in production with two instruments: BERMONIS and GFS-3000. Data recorded by BERMONIS were greater than those recorded by GFS-3000. However, the results show the same trend in variation. At nighttime, BERMONIS had greater measuring error in comparison to GFS-3000. This is due to the difference in performance of the main sensor, the CO_2_ sensor.

The GFS-3000 instrument showed a measuring error less than 0.2 ppm. Measurement accuracy of the CO_2_ sensor in BERMONIS was lower, with an error range of ± 3 ppm (VAISALA, manual). However, this measuring error has limited effect in high concentration measurement, for example during daytime. Indeed, as plants photosynthesis takes place in the daytime, it results in large difference in CO_2_ concentration between the inside and outside of the leaf chamber. At night, in the absence of light, plants cannot use CO_2_, and the CO_2_ concentration between the inside and outside of leaf chamber is thus limited.

Another factor is that the air flow in GFS-3000’s leaf chamber is more stable since GFS-3000 is installed with an impeller.


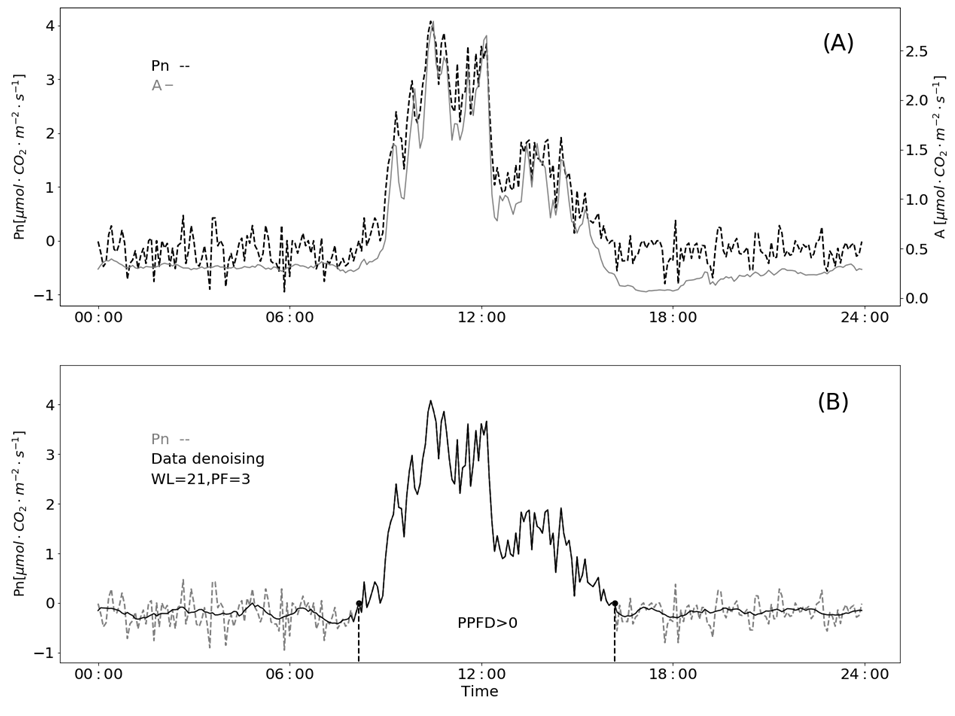


Fig. S-1 Diurnal measurement of net photosynthesis rate.

Where P_n_ is data recorded by BERMONIS (dotted line); A is data recorded by GFS-3000 (grey line).

In fact, low construction costs are also a feature of process instrumentation. Daytime is an important observation time for photosynthesis monitoring. Without affecting daytime monitoring, smoothing the night-time data can eliminate the influence of the nighttime measuring error on the overall value. In Fig S-2, a Savitzky-Golay filter method (window size: 35, polynomial order: 3), was applied to reduce nighttime measuring error. This method and its parameters are recommended for the further measurements when BERMONIS is used for process analysis.


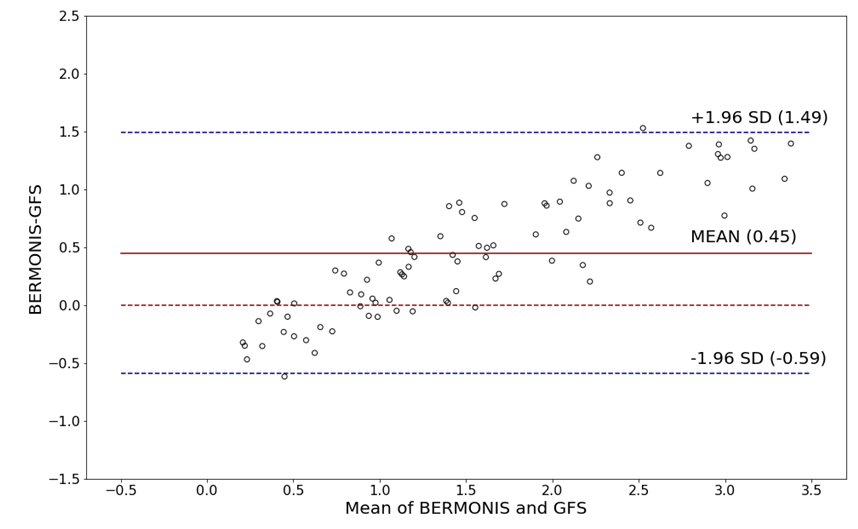


Fig. S-2 Bland-Altman plot of two gas exchange analyzers

**Consistency evaluation with Bland-Altman method**

As mentioned above, daytime data is primordial for monitoring significance, therefore daytime (PAR> 0) data is used to analyze the measurement consistency of the instruments. The Fig. S-2 displays a scatter diagram of the different plots against the averages of the two measurements. Horizontal lines are drawn at the mean difference, and at the limits of agreement. The mean difference is 0.45. 108 points are evenly distributed above and below the Mean line. The standard deviation (SD) of the differences is 0.53. The limits of agreement (LoA) defined as the mean difference ± 1.96 SD of differences is in the range of -0.59 to 1.49. From the figure only two points are out of LoA, which is 1.79% (<5%) of all points. This result demonstrates that the o instruments has good consistency and can be used interchangeably.
