# Supplementary material for: Crop Photosynthetic Performance Monitoring Based on a Combined System of Measured and Modelled Chloroplast Electron Transport Rate in Greenhouse Tomato
Source: Front Plant Sci. 2020 Jul 10;11:1038. doi: 10.3389/fpls.2020.01038 (PMC7381275; doi:10.3389/fpls.2020.01038)
Supplement: Supplementary file 3 [file DataSheet_3.docx]

**Supplementary Material C:**

**(Sub-models of Model I)**

The leaf net assimilation rate $A_{nL}$ is determined from the gross leaf assimilation rate $A_{gL}$ minus mitochondria respiration $R_{d}$.

$A_{nL}= A_{gL}-R_{d}$ (C-1)

$A_{gL}$ is determined from the negative exponential light-response curve (Goudriaan and van Laar, 1994).

$A_{gL}= A_{gmax}\cdot\left( 1-e^{-\frac{\varepsilon I_{abs}}{A_{gmax}}} \right)$(C-2)

$\varepsilon$ is the light use efficiency by photorespiration and $I_{abs}$ is the absorbed radiation.

$\varepsilon=\beta\frac{M_{CO2}}{4}\cdot\left( \frac{\max\left( C_{a},\Gamma^{*} \right)-\Gamma^{*}}{\max\left( C_{a},\Gamma^{*} \right)+2\Gamma^{*}} \right)$(C-3)

in which the number of e^-^ per fixed CO_2_ is 4, where *C_a_* is the CO_2_ concentration in the greenhouse, $M_{CO2}$ is the molar mass of CO_2_ and $\beta$ is the conversion factor from *J* to μmol e^-^, is equal to 1.6065 (Goudriaan and van Laar,1994).$\Gamma^{*}$ is the compensation concentration in the absence of dark respiration (Farquhar et al., 1980) is calculated by:

$\Gamma^{*}=\frac{K_{C}}{2K_{O}}\cdot O\cdot f_{OC}$ (C-4)

In which $O$ is the O_2_ partial pressure inside the stomata is 210 mbar. $f_{OC}$ is the ration of maximum oxygenation rate to maximum carboxylation rate, is assumed constant, 0.21. $K_{C}$ is the Michaelis Menten constants for Rubisco carboxylation (Eqn. B-5), and $K_{O}$ is the Michaelis Menten constants for Rubisco oxygenation (Eqn. B-6).

$K_{C}=K_{C25}\cdot e^{E_{C}\cdot\frac{T_{c}-T_{25}}{T_{c}\cdot R_{g}\cdot T_{25}}}$ (C-5)

$K_{O}=K_{O25}\cdot e^{E_{O}\cdot\frac{T_{c}-T_{25}}{T_{c}\cdot R_{g}\cdot T_{25}}}$ (C-6)

In which $K_{C25}$ is 310 μbar. The activation energy $K_{C}$ Rubisco carboxylation ($E_{C}$) is 59356 J mol^-1^.$K_{O25}$ is 155 mbar. The activation energy $K_{O}$ Rubisco oxygenation ($E_{O}$) is 35948 J mol^-1^ (Farquhar et al., 1980; Gijzen, 1994).

The maximum gross assimilation rate $A_{gmax}$ is calculated by:

$A_{gmax}=A_{nmax}+M_{CO2}\cdot R_{d}$ (C-7)

The leaf dark respiration rate $R_{d}$ is calculated by:

$R_{d}=R_{d25}\cdot e^{E_{D}\cdot\frac{T_{c}-T_{25}}{T_{c}\cdot R_{g}\cdot T_{25}}}$(C-8)

Where the dark respiration at 25℃ ($R_{d25}$) is 1.1μmol (CO_2_) m^-2^s^-1^. Activation energy $r_{D}$ dark respiration rate ($E_{D}$) is 66405 J mol^-1^.

The maximum (light saturated) net assimilation rate ($A_{nmax}$) is given by:

$A_{nmax}= \frac{A_{mm}+A_{nc}-\sqrt{{(A_{mm}+A_{nc})}^{2}-4\theta\cdot A_{mm}\cdot A_{nc}}}{2\theta}$ (C-9)

In which $\theta$ is a factor for the degree of curvature, is equal to 0.7. The maximum endogenous photosynthetic capacity $A_{mm}$ is given by:

$A_{mm}=\frac{M_{CO2}}{4}\cdot J_{max}$(C-10)

Where the maximum electron transport rate $J_{max}$ is given by:

$J_{max}=J_{max25}\cdot e^{E_{J}\cdot\frac{T_{c}-T_{25}}{T_{c}\cdot R_{g}\cdot T_{25}}}\cdot\frac{1+e^{\frac{s\cdot T_{25}-H}{R_{g}\cdot T_{25}}}}{1+e^{\frac{s\cdot T_{C}-H}{T_{c}\cdot R_{g}}}}$ (C-11)

Where the constant S is equal to 710 J mol^-1^K ^-1^ and H is equal to 220000 J mol^-1^. The CO_2_ limited rate $A_{nc}$ of net photosynthesis is given by:

$A_{nc}= \frac{\rho_{CO2}\cdot\frac{T_{0}}{T_{C}}}{R_{tot\_CO2}}\cdot\left( \max\left( C_{a},\Gamma\right)-\Gamma\right)$(C-12)

Where $\rho_{CO2}$is the ${CO}_{2}$ density at $T_{0}$. The total resistance to ${CO}_{2}$ diffusion $R_{tot\_CO2}$ is calculated by adding stomatal, boundary layer and carboxylation resistance.

$R_{tot\_CO2}= R_{s_{CO2}}+R_{b_{CO2}}+R_{c_{CO2}}$(C-13)

$R_{s_{CO2}}=1.6\cdot R_{s_{H2O}}$(C-14)

$R_{b_{CO2}}=1.37\cdot{Le}^{\frac{2}{3}} \cdot\frac{1174\sqrt{l_{f}}}{{(l_{f}\cdot\left| T_{l}-T_{a} \right|+207{va}^{2})}^{\frac{1}{4}}}$(C-15)

Where Le=0.89 is the Lewis number for water vapour in air, leaf width $l_{f}$=0.035 m and wind speed (in the greenhouse) va=0.09 m/s, $\left| T_{l}-T_{a} \right|$ is the difference of leaf and air temperature in K.

$R_{c_{CO2}}=\frac{K_{M}}{V_{Cmax}}\cdot\frac{\rho_{CO2 \frac{T_{0}}{T_{C}}}}{M_{CO2}}$(C-16)

Where the effective Michaelis Menten constant for carboxylation ($K_{M}$) is given by:

$K_{M}= K_{C}\cdot\left( 1+\frac{\rho_{O2i}}{K_{O}} \right)$(C-17)

The maximum carboxylation rate ($V_{Cmax})$ is calculated by:

$V_{Cmax}=V_{Cmax25}\cdot e^{E_{VC}\cdot\frac{T_{c}-T_{25}}{T_{c}\cdot R_{g}\cdot T_{25}}}$(C-18)
